# The Psychopathology of Worthlessness in Depression

**DOI:** 10.3389/fpsyt.2022.818542

**Published:** 2022-05-19

**Authors:** Phillippa Harrison, Andrew J. Lawrence, Shu Wang, Sixun Liu, Guangrong Xie, Xinhua Yang, Roland Zahn

**Affiliations:** ^1^Centre for Affective Disorders, Psychological Medicine, Institute of Psychiatry, Psychology and Neuroscience, King's College London, London, United Kingdom; ^2^Department of Psychology, Institute of Education, Hunan Agricultural University, Changsha, China; ^3^Key Laboratory of Psychiatry and Mental Health of Hunan Province, Mental Health Institute of the Second Xiangya Hospital, National Technology Institute of Psychiatry, Central South University, Changsha, China; ^4^National Service for Affective Disorders, South London and Maudsley NHS Foundation Trust, London, United Kingdom

**Keywords:** depression, self-blame, psychopathology, self-esteem, anhedonia, positive emotion, network analysis

## Abstract

**Background:**

Despite common dissatisfaction with the syndromic heterogeneity of major depression, investigations into its symptom structure are scarce. Self-worthlessness/inadequacy is a distinctive and consistent symptom of major depression across cultures.

**Aims:**

We investigated whether self-worthlessness is associated with self-blaming attribution-related symptoms or is instead an expression of reduced positive feelings overall, as would be implied by reduced positive affect accounts of depression.

**Methods:**

44,161 undergraduate students in Study 1, and 215 patients with current Major Depressive Disorder (MDD) and 237 age-matched healthy control participants in Study 2 completed the well-validated Symptom Check List-90. Depression-relevant items were used to construct regularized partial correlation networks with bootstrap estimates of network parameter variability.

**Results:**

Worthlessness co-occurred more strongly with other symptoms linked to self-blaming attributions (hopelessness, and self-blame), displaying a combined edge weight with these symptoms which was significantly stronger than the edge weight representing its connection with reduced positive emotion symptoms (such as reduced pleasure/interest/motivation, difference in edge weight sum in Study 1 = 2.95, in Study 2 = 1.64; 95% confidence intervals: Study 1: 2.6–3.4; Study 2: 0.02–3.5; Bonferroni-corrected *p* < 0.05).

**Conclusions:**

This confirms the prediction of the revised learned helplessness model that worthlessness is most strongly linked to hopelessness and self-blame. In contrast, we did not find a strong and direct link between anhedonia items and a reduction in self-worth in either study. This supports worthlessness as a primary symptom rather than resulting from reduced positive affect.

## Introduction

The defining symptoms of major depression are heterogenous and their pathophysiological role is elusive. Self-worthlessness/inadequacy is a consistent symptom of depressive disorders across cultures (1) and is more distinctive (2) than other consistent symptoms such as depressed mood which is shared across many diagnostic categories. Furthermore, low self-worth is one of the few reproducible predictors of future recurrence risk after remission of a depressive episode (3). It is therefore a good candidate for a so-called “primary symptom,” which was classically defined as one which is more closely related to the underlying pathophysiology (4). Recently, using network analytical approaches (5), there has been a rekindled interest in classical psychopathological questions about co-occurrence patterns of primary symptom candidates in current depression as clues to the underpinning neurocognitive disturbance and their pathophysiological validity. Here, we blend this usually descriptive approach with a formal statistical approach to investigate whether worthlessness is an expression of reduced positive feeling overall as the underpinning primary symptom or whether a reduced feeling of self-worth is independent of other positive feelings, suggesting lack of self-worth as an independent primary symptom. Answering this question is of fundamental importance to the pathophysiological understanding of major depressive disorder (MDD) and has important implications for the stratification of MDD in clinical trials as this may reveal dissociable subsyndromes that may respond differently to a particular type of treatment.

One of the most influential psychological models of MDD predicts that a combination of reduced positive affect and increased negative affect characterizes depression and this is supported by measures which sum up global scores across emotions irrespective of the entailed direction of blame or praise (6–8). Worthlessness, if conceptualized as a lack of positive feelings about oneself would therefore be predicted to be closely associated with a lack of positive feelings more generally manifesting in a lack of interest and pleasure, often referred to as “anhedonia.” In accordance with the Human Affectome Project, we use the term “feelings” here to describe complex subjective experiences which entail cognitive and emotional elements (9). On the other hand, feelings of worthlessness play a key role in the alternative revised learned helplessness model (10), which postulates that self-worthlessness is a result of overgeneralized self-blame and is strongly associated with hopelessness. The model purports that individuals attribute blame to themselves in an overgeneralised way that is internal, global and stable, which results in them feeling helpless, hopeless, worthless, as well as guilty and depressed for their perceived failings. Here, we probed the competing predictions of these models for the first time using a formal statistical test of the symptom network structure in a non-clinical and a clinical sample using the same well validated self-report measure (11).

The so-far largest transcultural study using validated observer-rated instruments to characterize individual symptoms in diagnosed depressive disorders to our knowledge found the feeling of inadequacy (including self-worthlessness) to be a consistent symptom of depression (1). In contrast guilt was only found in a subgroup of patients across cultures (12). Other studies, however, have reported a wide variation in the consistency of guilt/worthlessness which was most often reported as a single item following the Diagnostic Statistical Manual (DSM) (13). The frequency of guilt/worthlessness in current MDD was found to be between 20 (14, 15), 50% (16), and 70–80% (17, 18). DSM worthlessness was separately reported in another study as being present in 61% of current MDD patients (19). Guilt and low self-esteem were also found to be among the 36 transculturally ubiquitous symptoms of depression in a systematic review of qualitative studies (20). The discrepancy in reported frequencies of worthlessness and other symptoms is likely due to methodological as well as sampling differences. The semi-structured interviews for DSM were designed to provide reliable diagnoses rather than to assess single symptoms or the coherence of symptoms (21). As a consequence, the criterion threshold for different items on the DSM varies between symptoms rendering a direct comparison and analyses of symptom coherence invalid. In contrast, the Hopkins SCL-90 uses a comparable scaling for each symptom item (11) and its “feeling worthless” item was transculturally stable and associated with higher levels of depressive symptoms in traumatized non-clinical populations across a set of low- and middle-income countries (22).

A recent application of the novel method of network theory to the SCL-90 found worthlessness to be closely associated with “blaming yourself for things,” “feelings of guilt,” “feeling inferior to others” and “thoughts of ending your life” (23). The research found self-blame and guilt to be associated through a mutual connection with worthlessness. This indicated that worthlessness could play a role as predicted by the revised learned helplessness model, linked to guilt and self-blame. Furthermore, in an earlier smaller retrospective study, we found a group of self-blaming feelings including self-disgust/ contempt and guilt to closely co-occur with feelings of inadequacy, depressed mood and hopelessness, but affective rigidity and a lack of interest were equally closely associated and consistent (24). Therefore, despite links between worthlessness and self-blaming feelings, there is also evidence that worthlessness could be accounted for by reduced positive affect more generally. In contrast, a recent meta-analysis of network-based studies of depressive symptoms revealed worthlessness- and anhedonia-related items to be indirectly but strongly linked to the most central item of depressed mood, suggesting no direct link between worthlessness and anhedonia (25). This finding is, however, limited by the fact that self-blame and hopelessness could not be assessed, because most included studies used scales such as the standard Patient Health Questionnaire-9 which does not assess these separately, a limitation we have addressed in a recent modified version (26).

Network analysis provides an innovative method of growing popularity over the last decade (5) to understand the relationships between such symptoms of depression. The SCL-90 is a widely and transculturally validated self-report instrument to assess a broad range of psychiatric symptoms and includes subscales with depression-relevant symptoms (11). Here, we report two studies to investigate the symptom structure of depressive symptoms as measured on the SCL-90. Study 1 used a very large non-clinical sample to understand whether feelings of worthlessness are more closely related to the core symptoms of depression according to the revised learned helplessness model (guilt, self-blame, helplessness) or core symptoms of reduced positive affect (loss of interest, low libido, low energy). Network analysis was conducted in Study 1 to locate worthlessness in its network proximity to the learned helplessness subsyndrome and the reduced positive affect subsyndrome, respectively. Study 2 aimed to determine whether the network structure identified in Study 1 generalizes to a sample with a clinical diagnosis of current major depressive disorder and matched healthy controls (HC). The rationale for comparing these very different groups was to extract coherence of subsyndromes across different levels of severity and independent of the different sampling biases which affect any type of recruitment setting.

## Methods

### Ethics and Consent

The authors assert that all procedures contributing to this work comply with the ethical standards of the relevant national and institutional committees on human experimentation and with the Helsinki Declaration of 1975, as revised in 2008. All procedures involving human subjects were approved by the ethical review committees of Hunan Agricultural University (Reference: 2018089), the Second Xiangya Hospital (Reference: 2018S035), China and King's College London, UK (Reference: LRS-18/19-8404). Study procedures were explained to all participants, who provided written informed consent prior to assessment. Participation in the study was voluntary and participants did not receive any financial compensation. For study 1, the psychological health and education center administered the screening survey for mental health problems, with approval from the Ministry of Education to first year university students in October annually between 2005 and 2016. All participants provided informed consent before completing the survey.

## Study 1

### Participants

Using a multi-year consecutive cohort design, data were based on a routine Chinese Ministry of Health initiated mental health survey administered to first year university students every year between 2005 and 2016 by the psychological health and education centre at Hunan Agricultural University, Changsha. A total of 44,161 university students participated, with an average age of 18.53 (*SD* = 0.66), with 47.5% being female and 52.5% male. Supplementary Table 1 shows the number of participants by year of participation.

### Measures

The Symptom Check-List-90 (SCL-90) is a 90-item self-report symptom inventory designed to screen for a broad range of psychopathology. The Chinese adaptation of the SCL-90 was used in the present study (27). Each of the 90 items is rated on a 5-point Likert scale from 1 (“not at all”) to 5 (“extremely”). In the present study, the depression subscale items (13 items) and the guilt item (#89) were used to investigate our hypotheses.

### Procedures

The survey was completed electronically on a computer at the university and took approximately 15 min to complete. The health and education center staff explained the aim of the study to participants.

## Study 2

### Participants

Study 2 included *n* = 452 participants: 215 patients with current MDD from an inpatient clinic at the Second Xiangya Hospital (an additional 2 patients were excluded due to missing SCL-90 data) and 237 healthy control volunteers (HC), recruited via advertisements in the local community, matched for age and gender, but showing a slightly but significantly higher level in education (Supplementary Table 1).

Diagnoses of current MDD were made by an experienced psychiatrist (GRX) using the Structured Clinical Interview for the Diagnostic and Statistical Manual of Mental Disorders-IV [SCID-IV; 28]. Inclusion criteria for the MDD sample were: (1) current major depressive episode for ≥ 8 weeks to guard against including spontaneously remitting forms of MDD, (2) aged 18–45. Exclusion criteria were: (1) presence of another Axis I disorder including substance abuse, (2) a neurological disorder, (3) current or past psychotic features, and (4) currently receiving electroconvulsive therapy.

There were no specific inclusion criteria for the healthy control sample. Exclusion criteria for the HC sample were: (1) previous head injury, (2) cortisol or benzodiazepine medication in the previous 3 months, (3) a neurological disorder, (4) any comorbid mental illness, and (5) family psychiatric history.

### Measures

The Structured Clinical Interview for the Diagnostic and Statistical Manual of Mental Disorders-IV (SCID-IV) was administered by a psychiatrist to determine eligibility criteria for the MDD sample (28). The SCID is a semi-structured clinical interview used to assess Diagnostic Statistical Manual (DSM) mental disorder diagnoses. The DSM-IV was found to have good reliability for diagnosing MDD, *k* = 0.67. As in Study 1, the Chinese adaptation of the SCL-90 was used in the present study (27, 29).

The Chinese adaptation of the Zung Self-Rating Depression Scale, a standard self-report measure (27, 30) was used to assess depression severity in the MDD sample and to exclude healthy control participants with a score of ≥50 (Supplementary Table 3).

### Data Analysis

Statistical analyses were conducted using the Statistical Package for the Social Science (SPSS), version 24.0 and R Statistical Software. Particularly R and the bootnet package, v1.3 (31, 32) were used to construct regularized partial correlation networks with bootstrap estimates of network parameter variability. There was no missing data on any of the SCL-90 items.

The 13 depression subscale items of the SCL-90 were used for network construction as well as the “guilt” item which we considered a symptom of depression, although it was not included in the original subscale. Separate networks were constructed for two datasets (Study 1 and Study 2). The 14 5-point Likert items were treated as binary, contrasting low symptom scores (1–2; indicating absent or mild distress) against high symptom scores (3–5; indicating distress rated between moderate and severe). We binarized the Likert scale scores under the assumption that the distinction between at least moderate vs. mild or absent symptom levels would be more informative of clinically relevant symptoms. This threshold-based binarized approach aligns with our previous investigation into the psychopathology of depression using semi-structured interviews (24) and is also the standard way of defining the clinical relevance of symptom criteria on the structured clinical interview for DSM (21).

The eLasso method was used to construct the symptom network (33–35). In brief, node-wise ℓ_1_-regularized regression [glmnet, v2.0.18; (36)] was conducted over an evenly spaced range of 100 penalty parameters (λ), and optimal λ was determined using the Extended Bayesian Information Criterion (EBIC) (37) given the log-likelihood under the Ising model (33). The EBIC method requires setting a tuning parameter (0 ≤ γ ≤ 1) which, in effect, determines the contribution to the EBIC of a prior inversely proportional to network density. We use the recommended γ = 0.25 setting (35). Non-parametric, percentile method bootstrap confidence limits (10,000 samples) for network quantities of interest were obtained from bootnet software. Confidence width was 0.975 corresponding to a two-tailed alpha of 0.025.

Permutation tests were used to probe differences in network structure between the two datasets using the Network Comparison Test package for R (38). In brief, the data was pooled and study membership (Study 1 or Study 2) was permuted 10,000 times; each time networks for the two (permuted) study datasets were constructed as above. Permutation *p* values were then obtained to assess network invariance (38) along with the total network strength (i.e., sum of weights) and individual network edges. We adjusted the permutation-*p* values for individual network edges (*n* = 91) for multiple comparisons using the holm-bonferroni step-down procedure (39).

To test the primary hypothesis, summed eLasso edge weights were assessed between the probe symptom of worthlessness and reduced positive affect items and revised learned helplessness (RLH) items, respectively. The summed weights between worthlessness and reduced positive affect items (RPA) and between worthlessness and revised learned helplessness items were compared to identify which summed weights were greater. This was performed separately within each study dataset by calculating bootstrap 97.5% confidence intervals for the difference in the summed edge-weights (Δ_*RLH*−*RPA*_, see equations 1-3). Intervals excluding zero were described as statistically significant.


(1) $$\begin{array}{l} \sum w_{RLH}=w_{WL-SB}+w_{WL-HL}+w_{WL-GU}\# \end{array}$$

(2) $$\begin{array}{l} \sum w_{RPA}=w_{WL-LS}+w_{WL-LE}+w_{WL-NI}\# \end{array}$$

(3) $$\begin{array}{l} \Delta_{RLH-RPA}=\sum w_{RLH}-\sum w_{RPA}\# \end{array}$$

Where *w*_*A*−*B*_ is the network edge weight between symptoms A and B, see Table 1 for symptom codes. Pairwise edge differences between the RPA and RLH edges were also examined (total 9 edge pairs) with statistical significance determined when confidence intervals (adjusted for multiple testing with a Bonferroni-corrected alpha=0.05/18 ≈ 0.0028) excluded zero using bootnet's difference Test function (32).

Table 1Bootstrap tests for associations of worthlessness.

**Table:** 

**Primary test of hypothesis**							
		**Study 1**			**Study 2**		
**Quantity**	**Description**	**Observed**	**2.5%**	**97.5%**	**Observed**	**2.5%**	**97.5%**
Δ_*RLH*−*RPA*_	RLH – RPA	2.95*	2.59	3.37	1.64*	0.02	3.53
∑*w*_*RLH*_	Edge Sum RLH	3.69*	3.44	3.97	2.48*	1.38	3.96
∑*w*_*RPA*_	Edge Sum RPA	0.74*	0.49	0.98	0.84	0.00	1.86
**Component edges contained in the main comparison**							
		**Study 1**			**Study 2**		
**Quantity**	**Description**	**Observed**	**2.5%**	**97.5%**	**Observed**	**2.5%**	**97.5%**
*w*_*WL*−*GU*_	RLH: guilt	0.77*	0.57	0.98	0.90	0.00	1.80
*w*_*WL*−*HL*_	RLH: hopelessness	2.26*	2.11	2.40	1.28*	0.49	2.08
*w*_*WL*−*SB*_	RLH: self-blame	0.66*	0.51	0.82	0.30	0.00	1.07
*w*_*WL*−*LS*_	RPA: loss of sexual interest	0.09	0.00	0.25	0.20	0.00	0.88
*w*_*WL*−*LE*_	RPA: low energy	0.24*	0.07	0.40	0.00	−0.73	0.27
*w*_*WL*−*NI*_	RPA: no interest	0.41*	0.25	0.57	0.64	0.00	1.46

**Table:** 

**Difference between component edges**			
**Quantity**	**Description**	**Study 1**	**Study 2**
*w*_*WL*−*SB*_ − *w*_*WL*−*LS*_	self-blame-loss sexual interest	0.57* [0.26, 0.85]	0.10 [-0.99, 1.28]
*w*_*WL*−*SB*_ − *w*_*WL*−*LE*_	self-blame-low energy	0.42* [0.13, 0.77]	0.30 [-0.45, 2.05]
*w*_*WL*−*SB*_ − *w*_*WL*−*NI*_	self-blame-no interest	0.25 [-0.06, 0.55]	−0.34 [-1.64, 1.12]
*w*_*WL*−*HL*_ − *w*_*WL*−*LS*_	hopelessness-loss sexual interest	2.17* [1.86, 2.44]	1.08 [-0.34, 2.24]
*w*_*WL*−*HL*_ − *w*_*WL*−*LE*_	hopelessness-low energy	2.02* [1.73, 2.36]	1.28* [0.16, 3.17]
*w*_*WL*−*HL*_ − *w*_*WL*−*NI*_	hopelessness-no interest	1.84* [1.55, 2.16]	0.64 [-1.06, 2.16]
*w*_*WL*−*GU*_ − *w*_*WL*−*LS*_	guilt-loss sexual interest	0.69* [0.34, 1.04]	0.70 [-0.75, 2.06]
*w*_*WL*−*GU*_ − *w*_*WL*−*LE*_	guilt-low energy	0.53* [0.18, 0.91]	0.90 [0.00, 2.78]
*w*_*WL*−*GU*_ − *w*_*WL*−*NI*_	guilt-no interest	0.36* [0.03, 0.70]	0.26 [-1.30, 1.89]

*Values are observed edge weights and their differences [lower confidence interval (CI), upper CI], with CI widths calculated at a Bonferroni corrected alpha of 0.05 / 18. In both datasets, confidence intervals for the difference in the sum of weights excluded zero with greater values observed for the connections between worthlessness and the RLH items. Asterisks indicate where the confidence intervals exclude zero. Network edge weights (non-zero eLasso network weights) correspond to the regularized strength of the association between items adjusted for the association with all other items in the network. An association between items arises by direct co-occurrence across subjects whilst adjusting for indirect co-occurrence through other items in the network. WL, worthlessness; RPA, Reduced Positive Affect-related symptoms; RLH, Revised Learned Helplessness-related symptom items*.

## Results

### Frequency of Depression Subscale Items

Endorsement of items on the SCL-90 depression subscales for the student sample are displayed in Supplementary Figure 1, and for the MDD and HC sample in Supplementary Figure 2. In Study 1, “feeling lonely” was the most common item, followed by “worrying too much about things” and “blaming yourself for things.” In Study 2, “feeling low in energy or slowed down” and “worrying too much about things” were the most commonly endorsed symptoms in the HC sample. All symptoms were endorsed more frequently by the MDD sample than in Study 1 and the HC sample, with “feeling blue” and “feeling low in energy or slowed down” being the most common.

### Symptom Networks

**Figure 1:**
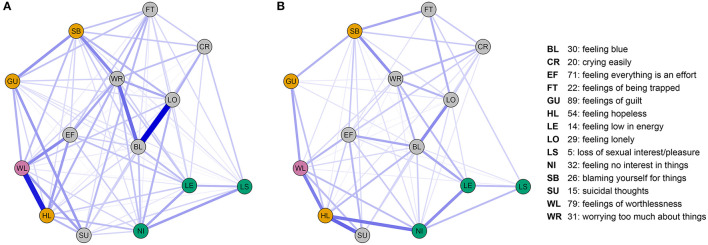
**(A)** SCL90 depression-relevant symptom network obtained from Study 1; **(B)** SCL90 depression-relevant symptom network obtained from study 2. Displayed networks comprise nodes (questionnaire items) and edges (non-zero eLasso network weights). Edges correspond to the regularized strength of the association between items adjusted for the associations between all other items in the network. Nodes are colored by their hypothesized role: worthlessness (pink), Reduced Positive Affect items (green), Revised Learned Helplessness items (yellow), other items (gray). Node positions are identical between studies to aid comparison of the networks. Node position was determined by a force-directed “spring” layout of the average weights taken over study 1 and study 2. Non-zero network edges are displayed in blue using a consistent size/transparency scale over the two networks.

We inspected the depressive symptom networks for direct (i.e., independent) associations with worthlessness in the non-clinical (Figure 1A) and clinical case-control (Figure 1B) studies. The network analyses revealed that worthlessness was co-occurring with hopelessness most strongly in both study samples whilst controlling for relationships with all other items. Suicidality was linked to hopelessness and worthlessness in both studies, but was linked to guilt directly only in Study 1. Loss of interest was linked to other reduced positive affect items (loss of sexual interest/pleasure, low energy) in both studies. Loss of interest was further directly associated with hopelessness in both studies, but not with worthlessness. Across both studies, worthlessness was associated not only with hopelessness, but also guilt and feeling that everything is an effort (i.e., inhibition of drive).

We further confirmed our primary hypothesis more formally by showing a significantly stronger association of worthlessness with self-blame, guilt and hopelessness relative to the reduced positive affect symptoms in both study samples by comparing the difference in the sum of weights (Table 1). When examining the contribution of individual symptom items, hopelessness emerged as significantly associated with worthlessness in both studies, whereas guilt and self-blame only showed a significant association in study 1. Weaker, but significant associations for worthlessness with low energy and interest were observed in study 1, but no associations with reduced positive affect items in study 2. We further examined the relative importance of connections with individual positive affect and learned helplessness symptoms by comparing relationships between worthlessness and three individual symptoms belonging to the learned helplessness, as well as three symptoms belonging to the reduced positive affect syndrome (9 edge-pair comparisons, Table 1). These comparisons revealed the association of worthlessness with hopelessness being significantly stronger than its association with low energy in both studies. Furthermore, for study 1 this analysis showed a stronger association of worthlessness with all individual revised-learned helplessness symptoms than with individual reduced positive affect items, except for the comparison of self-blame and loss of interest (Table 1).

### Consistency Across Study Samples

To determine whether the relationships between symptoms were similar in the non-clinical and clinical case control study, we compared the symptom networks of the depression items between study 1 and 2. Although, this revealed a greater number of non-zero network edges in Study 1 (85 / 91, 93% density) than Study 2 (62 / 91, 68% density), the average edge weights for non-zero edges were comparable (mean study 1: 0.54, mean study 2: 0.53). Permutation testing of between-study differences in both global and local (edge-related) network properties confirmed that there were no significant differences between study samples under permutation of study data. This was shown by a non-significant network structure invariance test (*p* = 0.501) and comparable total network strength (study1: 46.2, study2: 32.9, *p* = 0.97). In addition, no individual edges were found to significantly differ between the two study samples, the minimum *p* value was *p* = 0.819 (corrected, 0.009 uncorrected), which was for the connection “blue-lonely” [or “BL-LO”].

### Consistency Across Modeling Methods

To confirm our results were robust to our binary modeling choice, we repeated our key analyses using the ordinal raw SCL-90 item scores (employing eBICglasso in R-software). We were able to replicate our key findings using these raw scores. The difference in sum of edges favored revised learned helplessness items and excluded zero in the confidence intervals: RLH > RPA = +0.482 [0.453, 0.511] for study 1, and RLH > RPA = +0.545 [0.257, 0.732] for study 2. Further the permutation tests of network invariance between study 1 and study 2 networks were likewise non-significant 0.158, *p* = 0.25 with comparably non-significant total (*p* = 0.267) and individual edge strength comparisons (minimum *p* = 0.09).

Taken together, this shows the consistency of symptom relationships across study samples with apparent differences being attributable to the differing performance of the network model with increasing sample size–large samples require less regularization and so find more non-zero edges and larger weights.

## Discussion

As predicted by the revised learned helplessness model of depression (10), worthlessness was significantly more closely associated with core symptoms of overgeneralised self-blaming attributions which include hopelessness and excessive self-blame than with other symptoms of reduced positive affect (40) linked to anhedonia. To our knowledge, this is the first direct examination of these alternative hypotheses using network analytical methods as applied to psychopathology.

As Jaspers, the founder of phenomenological psychopathology, noted on the analyses of symptom complexes (4), there are different aspects of the relation of symptoms within a symptom complex: (1) frequency of symptom co-occurrence, (2) coherence of symptoms by being related to a common aspect or function, and (3) primary symptoms caused by the aetiopathogenetic process and secondary symptoms emerging from these in an understandable way. The second aspect, that of symptom coherence, has been emphasized by Carl Schneider (as reviewed by Karl Jaspers) arguing about symptoms: “Their connectedness must be due to a normal complex of psychic function, which complex has been affected by the illness.” As we have previously stated (41), at the time of Jaspers, a lack of knowledge about neurobiologically valid models of many higher cognitive functions hampered the success of this approach. Here, we have used a combination of modern network analytical tools with cognitive models of MDD to translate cognitive into psychopathological hypotheses, an approach needed to refine and enrich our current diagnostic systems (42).

Our finding that worthlessness is independent of reduced positive affect, once its non-specific relationships with all depressive symptoms have been adjusted for, further consolidates its role as a potential primary symptom of depression (24, 41) given that feelings of worthlessness/inadequacy are consistent across cultures (1) and subtypes of depression (43). Although the frequency of worthlessness in our current MDD group was lower than in the WHO collaborative study (1) using observer-rated measures, this is to be expected in that self-report scales do not allow for different subjective variations in which a symptom is experienced and labeled. For example, observer-rated psychopathology interviews, we have previously used to show a high frequency of worthlessness/inadequacy in MDD (24, 44), use prompt questions for feelings of worthlessness/inadequacy which include those arising from experiences of achievement-related and interpersonal failure. The former being more relevant for people striving for autonomy and the latter more for sociotropic individuals (45), who we have recently shown to differ with regard to the functional neuroanatomy of self-blame-related emotions (46).

Our finding of loss of interest/pleasure as strongly connected to hopelessness, whilst also being strongly associated with the other two symptoms putatively linked to low positive affect (loss of sexual interest and low energy) suggests that although partly independent, the learned helplessness subsyndrome is linked to the reduced positive affect subsyndrome and this association probably arises from more severe and melancholic forms of depression which are characterized in the DSM by a combination of anhedonia and self-blame (21). Our findings, however, suggest that rather than using anhedonia as a necessary and self-blame as an accessory criterion for the melancholic subtype as is done in DSM, these should be conceived of independent subsyndromes which should not be placed into a hierarchical relationship.

## Limitations

On a more cautionary note, one needs to discuss the lack of information about co-morbidity and treatment history in our clinical sample as well as the sole reliance on self-report measures of psychopathology. The latter is an inherent weakness of our study that sought to identify consistent symptom profiles across a very large non-clinical and a moderately large clinical sample as it would not have been feasible to collect gold standard observer-rated measures of psychopathology, which we have used in a previous smaller sample (24). The slightly higher education level in the control group in Study 2 is unlikely to have affected our overall results as our primary conclusion does not rely on group comparison and rather on network structure across groups representing a broad range of different symptoms and severity. We were also unable to determine the effects of psychosocial factors on symptom profiles in this study, but these are unlikely to have affected the covariance of different symptoms and thereby the structure of the network. Future longitudinal studies are needed to determine whether the network structure of worthlessness identified here is specific for the depressed state or may also be detected outside of symptomatic states, reflecting vulnerability traits.

## Conclusions

Our results confirm the prediction of the revised learned helplessness model that worthlessness is most strongly linked to hopelessness and self-blame. Contrary, to the predictions derived from the reduced positive affect model of depression, we did not find a strong and direct link between reduced pleasure, interest and motivation/energy with a reduction in self-worth. These symptom relationships were consistent across non-clinical and clinical study samples which argue for their generalizability. This confirms previous work showing that MDD is associated with a more specific pattern of emotional disturbances than an overall decrease in positive and increase of negative affect (47).

## Data Availability Statement

The data used to support the findings of this study is available from the corresponding author upon request. The analysis code is publicly available (https://github.com/AndrewLawrence/PsychopathologyOfWorthlessness). The correlation coefficients underpinning the network analyses can be found in the supplementary matrices worksheet accompanying Supplementary Figure 3.

## Ethics Statement

The studies involving human participants were reviewed and approved by King's College London, UK (Reference: LRS-18/19-8404). The patients/participants provided their written informed consent to participate in this study.

## Author Contributions

PH wrote the draft of the manuscript. AL analyzed the data. SW and SL contributed to the data collection. GX diagnosed patients. XY managed the study and interpreted the data. RZ generated the idea, supervised the analysis, and revised the manuscript. All authors significantly contributed to and have approved the final manuscript.

## Funding

AL and RZ were funded by the National Institute for Health Research (NIHR) Biomedical Research Centre at South London and Maudsley NHS Foundation Trust and King's College London. XY was funded by a King's College London–KC Wong Fellowship. PH's post was funded from the National Institute for Health Research (NIHR) research for patient benefit (RfPB, grant reference: PB-PG-0416- 20039).

## Author Disclaimer

The views expressed are those of the authors and not necessarily those of the NHS, the NIHR, or the Department of Health.

## Conflict of Interest

The authors declare that the research was conducted in the absence of any commercial or financial relationships that could be construed as a potential conflict of interest.

## Publisher's Note

All claims expressed in this article are solely those of the authors and do not necessarily represent those of their affiliated organizations, or those of the publisher, the editors and the reviewers. Any product that may be evaluated in this article, or claim that may be made by its manufacturer, is not guaranteed or endorsed by the publisher.
