# Supplementary material for: The Psychopathology of Worthlessness in Depression
Source: Front Psychiatry. 2022 May 19;13:818542. doi: 10.3389/fpsyt.2022.818542 (PMC9160466; doi:10.3389/fpsyt.2022.818542)
Supplement: Supplementary file 1 [file Data_Sheet_1.docx]

**SUPPLEMENTARY MATERIALS**

**The psychopathology of worthlessness in depression**

Phillippa Harrison^1^; Andrew J Lawrence^1;^ Shu Wang^2^; Sixun Liu^2^; Guangrong Xie^3^;

Xinhua Yang^1^*; Roland Zahn^1, 4^

*^1^ Centre for Affective Disorders, Psychological Medicine, Institute of Psychiatry, Psychology & Neuroscience, King’s College London, London, UK.*

*^2^ Department of Psychology, Institute of Education, Hunan Agricultural University, Changsha, Hunan, China.*

*^3^ Mental Health Institute of the Second Xiangya Hospital, National Technology Institute of Psychiatry, Key Laboratory of Psychiatry and Mental Health of Hunan Province, Central South University, Changsha, Hunan, China*

*^4^ National Service for Affective Disorders, South London and Maudsley NHS Foundation Trust, London, UK*

**Correspondence should be addressed to:*

*Xinhua Yang,*

*Centre for Affective Disorders, Psychological Medicine, Institute of Psychiatry, Psychology & Neuroscience, King’s College London, London, UK.*

*Main Building, 3rd floor, Room E3.23, SE5 8AF*

*Tel. /fax: 07542563927*

*Email: xinhua.yang@kcl.ac.uk*

**Supplementary Figure 1| Study 1 - frequencies (%) of depressive symptoms**


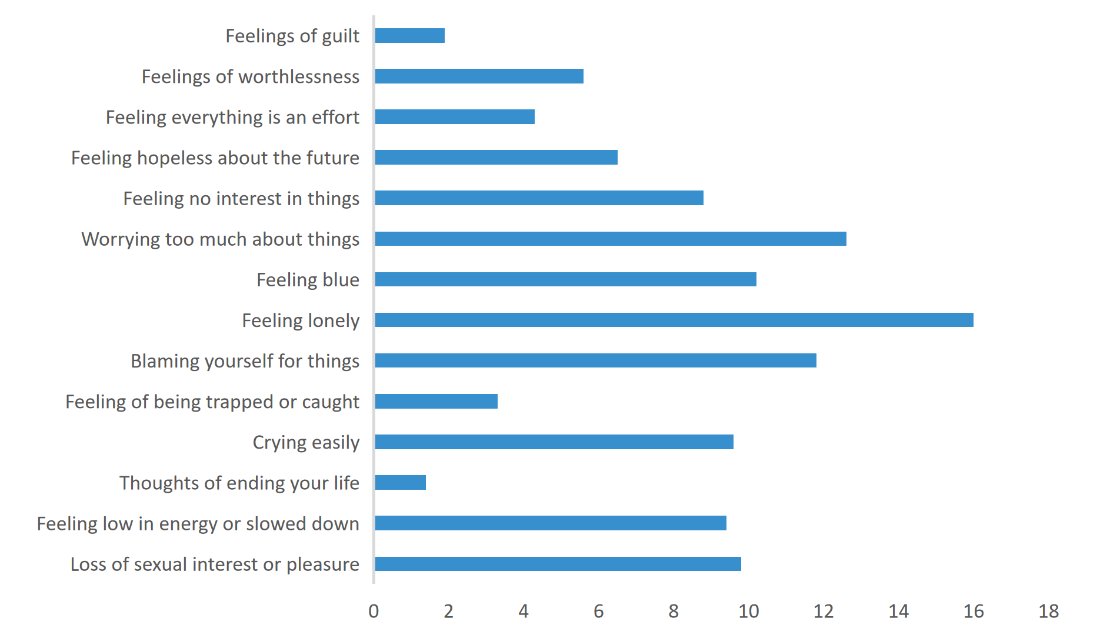


**Supplementary Figure 2| Study 2- frequencies (%) of depressive symptoms**

**
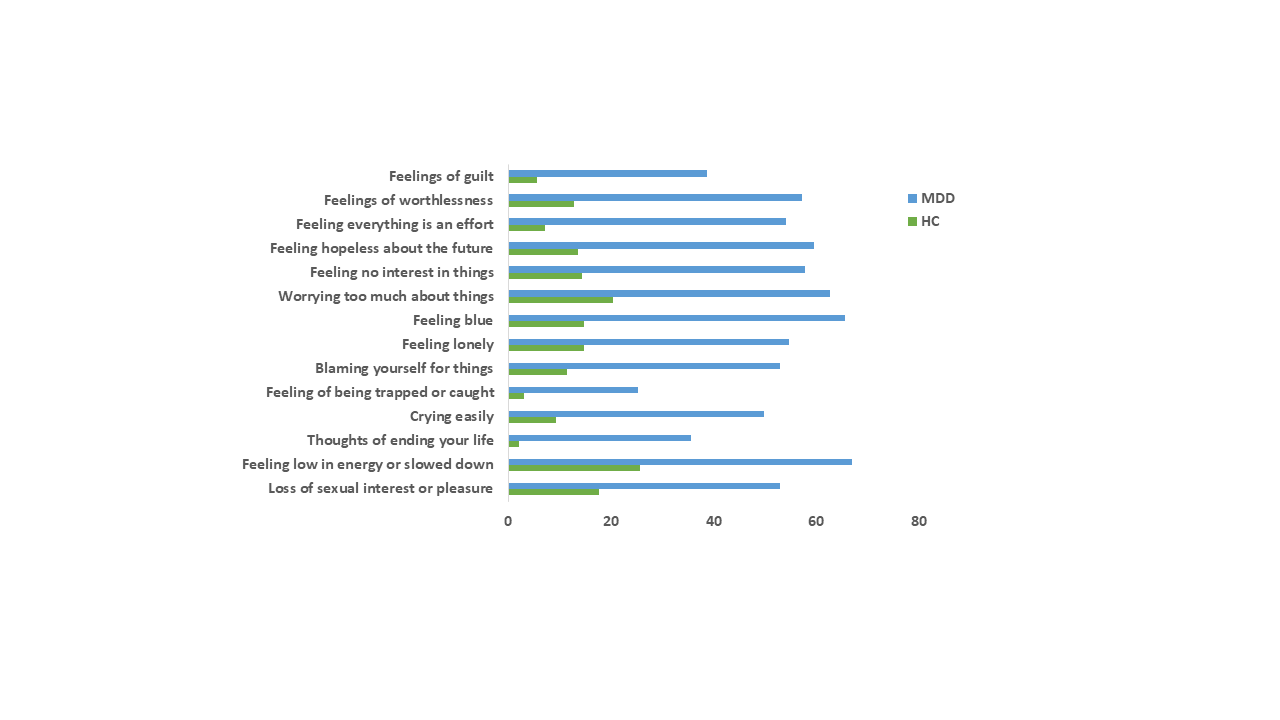
**

**Supplementary Figure 3| Matrix display of network coefficients**


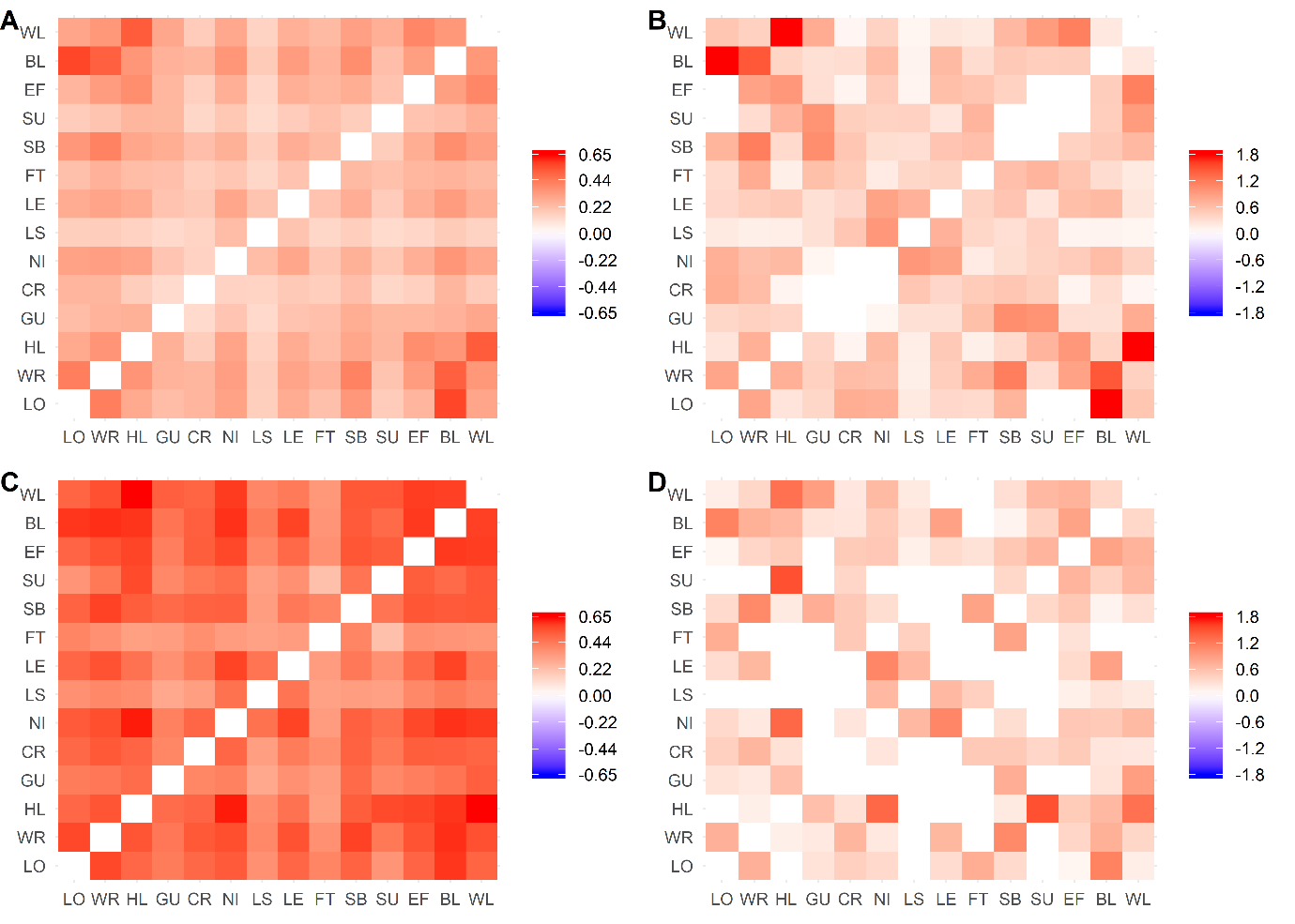


*Matrices are displayed for data from Study 1 (A, B; top row) and Study 2 (C, D; bottom row). For reach study the unadjusted phi coefficient matrix is displayed (A, C; left column) alongside a matrix of the eLasso coefficients (B,D; right column). A single colour scale is used in each column. In B edge weights greater than 99^th^ percentile (+1.8) are windsorised for display purposes. Items are ordered according to a dendrogram on panel B data. For item codes see key to Figure 1. The numerical values of these four matrices are included in the supplementary matrices excel file.*

**Supplementary Table 1 | Study 1 student sample characteristics**

| Year | Number of Participants |
| --- | --- |
| 2005 | 5579 |
| 2006 | 2679 |
| 2007 | 3463 |
| 2008 | 3042 |
| 2009 | 3697 |
| 2010 | 3027 |
| 2011 | 2202 |
| 2012 | 4478 |
| 2013 | 3463 |
| 2014 | 1990 |
| 2015 | 5204 |
| 2016 | 5337 |

*n=44161, 47.5% Female, age: mean=18.53, standard deviation=.66*

**Supplementary Table 2| Study 2 sample characteristics**

|  | Gender  (% female) | Age  (*M (SD))* | Years of education  (*M (SD))* | Zung Depression Scale  (*M (SD))* |
| --- | --- | --- | --- | --- |
| MDD | 52.5 | 30.25 (9.23) | 13.75 (2.66) | 52.92 (9.96) |
| HC | 53.2 | 29.11 (8.76) | 14.38 (2.78) | 38.07 (9.08) |

n = 217 MDD and n = 237 HC participants took part. MDD and HC groups were of comparable gender (χ^2^ (1)=.02, *p*=.89) and age composition (*t*(452)=-1.3, *p*=.17). As expected, there was a statistically significant difference between the MDD and HC groups on the Zung Self-Rating Depression Scale score, *t* (452) = -16.62, *p* < .001. The HC group showed a slightly but significantly higher education level (*t*(452)=2.5, *p* = .01). MDD= current major depressive disorder; HC = healthy control participants.

**Supplementary Table 3| Study 2 SCL-90 subscale scores**

|  | Somatization | OC | IS | Depression | Anxiety | Hostility | PA | PI | Psychoticism | Additional items |
| --- | --- | --- | --- | --- | --- | --- | --- | --- | --- | --- |
| MDD mean (SD) | 2.11  (.82) | 2.69 (.92) | 2.39 (.95) | 2.76  (.98) | 2.49 (.94) | 2.28 (.98) | 2.03 (.88) | 2.11 (.88) | 2.20 (.82) | 2.55 (.87) |
| HC mean (SD) | 1.49  (.43) | 1.92 (.55) | 1.72 (.56) | 1.66  (.50) | 1.55 (.51) | 1.63 (.57) | 1.35 (.43) | 1.55 (.50) | 1.49 (.43) | 1.62 (.52) |
| MDD Median (IQR) | 2.04 (1.42-2.67) | 2.70 (1.90-3.40) | 2.33 (1.67-3.00) | 2.85 (1.96-3.62) | 2.40 (1.70-3.30) | 2.00 (1.42-3.08) | 1.71 (1.29-2.57) | 2.00 (1.33-2.67) | 2.10 (1.50-2.80) | 2.57 (1.86-3.29) |
| HC Median (IQR) | 1.33 (1.17-1.75) | 1.80 (1.60-2.20) | 1.67 (1.33-2.00) | 1.62 (1.31-1.92) | 1.50 (1.20-1.80) | 1.50 (1.17-1.83) | 1.14 (1.00-1.57) | 1.50 (1.17-1.83) | 1.40 (1.15-1.70) | 1.57 (1.29-1.86) |
| *U* | 14003.00* | 12945.00* | 14637.50* | 9330.00* | 10229.00* | 15391.00* | 12585.00* | 16134.50* | 11885.50* | 9973.00* |
| *Z* | -8.34 | -9.15 | -7.94 | -11.74 | -11.10 | -7.42 | -9.48 | -6.89 | -9.92 | -11.30 |

N = 217 (MDD) (N = 216 for somatization subscale), N = 237 (HC); OC = obsessive compulsive; IS = interpersonal sensitivity; PA = phobic anxiety; PI = paranoid ideation, IQR=Interquartile range. SD=standard deviation. *=*p* <.0001. Mann-Whitney U tests were conducted as the SCL-90 subscales were not normally distributed (Kolmogorov-Smirnov test p< .001). MDD = major depressive disorder, HC = Healthy control.
